# Cerebellar vermis joins the brain’s social network

**DOI:** 10.1038/s42003-023-05673-6

**Published:** 2023-12-21

**Authors:** Viviane M. Saito

**Affiliations:** Memory Research Unit, Okinawa Institute of Science and Technology Graduate University (OIST), Okinawa, Japan

## Abstract

The cerebellum is more than just motor control: over the past 30 years, the notion that the “little brain” participates in cognitive functions and emotional response has grown to encompass social-related behaviors. Chao et al. bring to light the role of the cerebellar vermis in orchestrating a specific component of social memory in mice.

**Figure:**
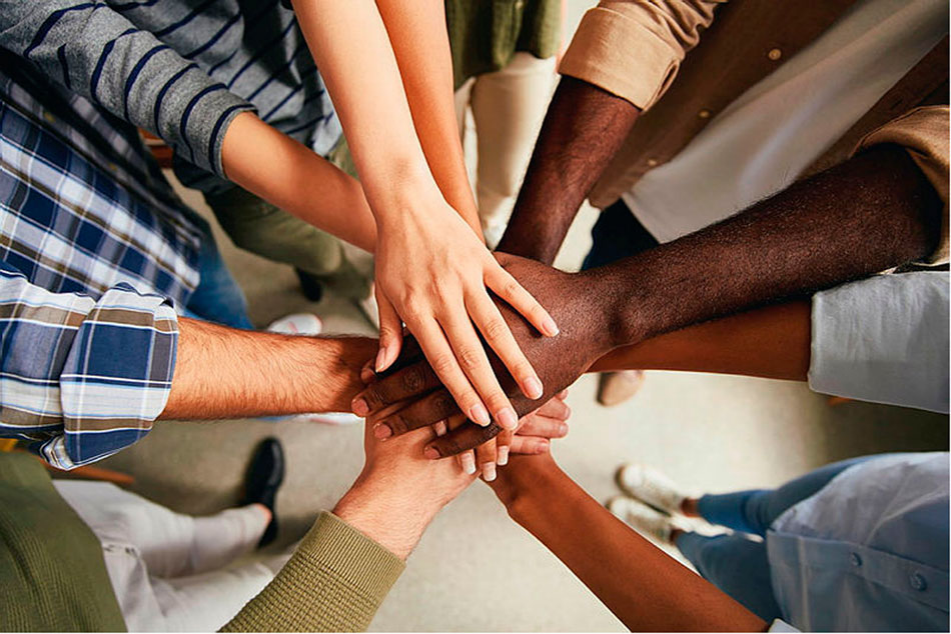
Kostiantyn, stock.adobe.com

Social behaviors involve a range of interactions crucial for the survival of individuals and species. These behaviors include various forms of communication, cooperation, and affiliative interactions within social groups. A critical aspect of social behaviors is social recognition memory (SRM), which involves recognizing familiar individuals to form and maintain social bonds. SRM typically consists of three stages: encoding, storage, and retrieval. During encoding, an individual perceives and processes social cues from conspecifics. Subsequently, these memories are stored and consolidated, allowing for the recognition of familiar individuals during later encounters. Finally, retrieval enables recalling stored information, facilitating appropriate social responses, and reinforcing social bonds. Understanding the neural substrates underlying social behaviors is particularly important in the context of various neuropsychiatric and neurodevelopmental disorders. For instance, in autism spectrum disorder (ASD) and other neuropsychiatric conditions, social behaviors are impaired, reflecting abnormal features of the social brain—a network of brain structures responsible for those behaviors. As part of the social brain, we can list the medial prefrontal cortex, anterior cingulate cortex, hippocampus, and amygdala. Now the cerebellar vermis joins this social team as a putative organizer of the neural matrix subserving SRM.

In a recent *Nature Communications* paper, Chao et al.^1^ use chemogenetic and optogenetic techniques to recapitulate the dysregulation of feedforward inhibition of Purkinje cells by molecular layer interneurons (MLI), seen in ASD patients and animal models. The authors exposed adult mice of both sexes to a battery of behavioral tests while chemogenetically increasing MLI excitability in specific lobules of the cerebellar vermis. These tests aimed to assess the animals’ ability to recognize a conspecific, while monitoring whether the experimental manipulation of the vermis would hamper motor activity or novelty-seeking behavior. The results from such a comprehensive approach show that vermis-disrupted animals displayed specific low social recognition scores without any abnormal anxiety levels, locomotion, exploratory motivation, social approach, or object recognition. Next, the researchers optogenetically stimulated MLIs during different sessions of the social recognition test. They observed that mice under MLI stimulation failed to discern between a complete stranger and a mouse they met in a previous session. Notably, this effect was not generalized to non-social recognition memory—they could perfectly recognize a novel object never seen before. This observation establishes direct evidence that the vermis constitutes a key player in the retrieval (or consolidation) of social information. Additionally, disrupting the tightly controlled local vermal circuitry perturbs the social brain network. The authors quantified c-Fos expression in 24 brain regions to identify neurons active during the social recognition test, with or without MLI stimulations. The c-Fos results together with graph theoretical analysis and neuroanatomical tracing revealed the complexity of the anatomical and functional connections between the cerebellum and the corticolimbic system that support SRM.

Overall, the study by Chao et al.^1^ is a timely effort to demonstrate the substantial participation of the cerebellum in the brain circuitry responsible for social memories, far beyond motor control. This research provides a first step in identifying the circuit basis for cerebellar involvement in social behaviors, and lays the groundwork for further developments in this field.
